# Proteomic Study of Aqueous Humor and Its Application in the Treatment of Neovascular Glaucoma

**DOI:** 10.3389/fmolb.2020.587677

**Published:** 2020-10-08

**Authors:** Mengxi Yu, Feng Xie, Xiang Liu, Haidan Sun, Zhengguang Guo, Xiaoyan Liu, Wei Li, Wei Sun, Ying Wang, Chengyan He

**Affiliations:** ^1^China–Japan Union Hospital of Jilin University, Changchun, China; ^2^Shanghai AB Sciex Analytical Instrument Trading Co., Ltd., Shanghai, China; ^3^Core Facility of Instrument, Institute of Basic Medical Sciences, Chinese Academy of Medical Sciences, School of Basic Medicine, Peking Union Medical College, Peking, China; ^4^Department of Ophthalmology, The Second Hospital of Jilin University, Changchun, China

**Keywords:** proteome, aqueous humor, neovascular glaucoma treatment, conbercept, vascular endothelial growth factor receptor

## Abstract

Aqueous humor (AH) proteins are involved in many physiological and pathological processes of the eye. The proteome analysis of AH is important to understand its physiological and pathophysiological functions. In the present study, AH samples obtained from 21 cataract volunteers were pooled together. After high-pH RPLC offline separation, the pooled sample was analyzed by LC-MS/MS to provide a comprehensive profile of AH proteome. The function analysis was provided by the GO and IPA annotation. In order to determine whether the AH proteome can reflect the pathophysiological changes of the disease, DIA technology was used to analyze the AH samples obtained from three neovascular glaucoma (NVG) patients (six samples) before and after drug treatment. The differential proteins were validated by PRM technology in an independent group (14 samples). In the AH proteome database, 802 proteins were identified, and 318 proteins were identified for the first time. Furthermore, 480 proteins were quantified based on the peak intensity-based semiquantification (iBAQ), which ranged by approximately 7 orders of magnitude. These proteins are primarily involved in immunity- and inflammation-related pathways. The differential AH proteomic analysis in NVG treatment revealed that the AH proteome can reflect the pathophysiological changes of drug treatment. Angiogenesis and thrombus coagulation progression are deeply involved in NVG treatment. The present experiment provided a comprehensive AH proteome analysis and expanded the profile of human AH proteome. The differential AH proteomic analysis of NVG treatment indicated that AH proteome can reflect the pathophysiological changes in drug intervention.

## Introduction

Aqueous humor (AH) is a transparent liquid secreted by the ciliary epithelium. It is mainly composed of 99.9% water, and trace amounts of sugar, vitamins, protein, and other factors (Macknight et al., 2000). The functions of AH are to maintain intraocular pressure, nourish the avascular cornea and lens, and remove metabolic waste (Bill, 1975). It plays an important role in the pathogenesis and progression of ophthalmic disease (Kliuchnikova et al., 2016). The analysis of the proteome in AH is important to understand the physiology and changes evoked by pathological situations, especially in posterior disorders.

In the past decade, several groups have provided the proteomic analysis of AH. Chowdhury et al. (2010) were the first to use LC-MS/MS technology to analyze the general profile of human AH proteins. They identified 354 proteins (Chowdhury et al., 2010). Murthy et al. (2015) used LC-MS/MS to identify 763 proteins in AH samples obtained from 240 cataract patients in 2015. In the proteomic study of AH in 24 patients with cataract and high myopia after the glaucoma/vitrectomy surgery in 2015, Ji et al. (2015) identified 445 proteins in AH. Adav et al. (2019) used LC-MS/MS to analyze the AH proteome and identified 834 proteins in the AH of cataracts.

In recent years differential AH proteomic analysis has been applied for ophthalmic diseases. Je-Hyun Baek et al. used SWATH-MS technology to quantify differentially expressed proteins in the AH between patients with drusen and reticular pseudodrusen (RPD) of age-related macular degeneration (AMD). In three newly discovered dry AMD-related proteins, LUM and KERA were upregulated in both RPD and soft drusen, and these are correlated to the extracellular matrix organization (Baek et al., 2018). Chiang SY et al. used MALDI-TOF MS and identified 11 AH proteins with a pathophysiological role in diabetic retinopathy. These proteins are linked with biological networks associated with nutrition transport, microstructure reorganization, angiogenesis, antioxidation, and neuroprotection (Chiang et al., 2012).

Previous studies have provided several AH proteome database analyses and disease differential analyses. With the development of proteomic techniques, a more comprehensive analysis should be provided to understand the function of AH. Furthermore, previous disease proteomic studies have revealed that AH can be an effective approach to identify disease differential proteins. However, more work is needed to determine whether these can reflect the disease pathophysiological change in treatment interventions.

In the present study, the investigators attempted to establish a comprehensive profile of AH proteome with offline 2D-LC/MS/MS analysis and provide a detailed proteomic functional annotation through the GO and IPA analysis. In addition, the investigators conducted a differential proteomic analysis of neovascular glaucoma (NVG) treatment with conbercept. The primary cause of NVG was the formation of new blood vessels above the iris, which block the outflow of AH and increase intraocular pressure (IOP) (Barac et al., 2015). Conbercept is a recombinant fusion protein designed as a VEGF receptor decoy. It can significantly inhibit neovascularization (Wang et al., 2013). In the present study, differential AH proteomic was used to investigate the proteomic changes in NVG treatment. The present study might benefit the understanding of AH proteome and accelerate the application of AH to clinical research.

## Materials and Methods

### Ethical Approval

Prior to study enrollment, all volunteers were given a verbal explanation of the study, and each participant provided a signed informed consent. The consent procedure and the research protocol for the present study were approved by the Ethics Committee for Human and Animal Research in Peking Union Medical College. The study methodologies conformed to the standards set by the Declaration of Helsinki.

### Reagents and Instruments

Dithiothreitol (DTT), iodoacetamide (IAM), formic acid, trifluoroacetic acid, ammonium bicarbonate, and HPLC-grade acetonitrile (ACN) were purchased from Sigma (St. Louis, MO, United States). Sequencing-grade trypsin was purchased from Promega.

A high-pH RPLC column (4.6 mm × 250 mm, Xbridge C18, 3 μm) and Orbitrap Fusion Lumos tribrid (Thermo Scientific, Bremen, Germany) coupled with an EASY-nLC 1000 was used for the MS analysis in the DDA-MS and DIA-MS modes.

A TripleTOF 5600 mass spectrometer from AB Sciex (Framingham, MA, United States) and an ACQUITY UPLC system from Waters (Milford, MA, United States) were used.

### Clinical Materials

The AH samples for dataset establishment were obtained from 21 cataract patients during surgery. The average age of these patients was 69.23 ± 12.5 years old, and the median age was 47 years old. A total of 20 AH samples from 11 patients were collected at 3 days before/7 days after the conbercept treatment during surgery to monitor the proteomic response after treatment of NVG (detailed clinical data in Supplementary Table S1). These investigators divided these randomly into two groups: the test group (three patients, six samples: three before treatment, three after treatment) and the validation group (11 patients, 14 samples: seven before treatment, seven after treatment). A week after treatment, the gonioscopy revealed a significant decrease in neovascularization in all posttreatment patients.

Each sample was approximately 50–200 μl and aspirated from the anterior chamber using a 26 needle at 3 days before/7 days after the conbercept treatment. After collection, the AH samples were immediately centrifuged at 2,500 × *g* at 4°C for 10 min to remove the cellular components and debris, and the supernatants were stored at -80°C for further analysis.

### Protein Extraction

The AH samples were precipitated overnight using three times the volume of ethanol at 4°C. Then, after centrifugation at 10,000 × *g* for 30 min, the pellets were resuspended in lysis buffer (7 M of urea, 2 M of thiourea, 0.1 M of DTT, and 5 mM of Tris). The protein concentration of 21 samples for AH proteome were pooled together and determined by spectrophotometry based on the Bradford method.

### Protein Digestion

The pooled sample was digested using a filter-aided sample preparation (FASP) method that was previously described by Gravett et al. (2004). The protein samples (200 μg) were reduced with 20 mM of DTT at 95°C for 5 min and carboxyamidomethylated with 50 mM of IAM at room temperature in the dark for 45 min. Trypsin (4 μg) in 25 mM of NH_4_HCO_3_ was added to each protein sample and incubated at 37°C overnight. After digestion, the resulting peptides were desalted on a Waters Oasis C18 solid-phase extraction column and lyophilized for HPLC separation.

### Offline HPLC

The peptides were separated with a high-pH RPLC column (4.6 mm × 250 mm, Xbridge C18, 3 μm). The sample was loaded onto the column in buffer A1 (H_2_O, pH 10). The elution gradient was 5-30% buffer B1 (90% ACN, pH 10; flow rate, 1 ml/min) for 30 min. The eluted peptides were collected at one fraction per minute. After this was lyophilized, the 30 fractions were resuspended in 0.1% formic acid for LC-MS analysis.

### LC/MS/MS

Orbitrap Fusion Lumos tribrid (Thermo Scientific, Bremen, Germany) coupled with the EASY-nLC 1000 was used for the MS analysis in the DDA-MS and DIA-MS modes. The digested peptides were separated on an RP C18 self-packing capillary LC column (75 μm × 100 mm, 3 μm). The eluted gradient was 5–30% buffer B2 (0.1% formic acid, 99.9% ACN; flow rate, 0.3 μl/min) for 60 min.

For the generation of the spectral library, the 30 fractions from RPLC were analyzed in DDA mode. The parameters were set as follows: the full scan was acquired from m/z 350–1,500 with a resolution of 60,000; the cycle time was set to 3 s; the auto gain control (AGC) was set to 1e6, and the maximum injection time was set to 50 ms; charge state screening (including precursors with + 2 to + 5 charge state) and dynamic exclusion (exclusion duration 30 s). MS/MS scans were performed at a resolution of 15,000, with an isolation window of 1.6 Da and collision energy at 32% (HCD). T, the AGC target, was set to 5e4, and the maximum injection time was 30 ms.

Each individual sample was analyzed in DIA mode. For MS acquisition, the variable isolation window DIA method with 38 windows was developed. The specific window lists were constructed based on the DDA experiment of the pooled sample. According to the precursor m/z distribution of pooled sample, the precursor ion number was equalized in each isolation window. The full scan was set at a resolution of 120,000 over the m/z range of 400–900, followed by DIA scans with a resolution of 30,000 (HCD collision energy: 32%; AGC target: 1e6 and maximal injection time: 50 ms). The MS data file can be freely downloaded at iProX (Integrated Proteome resources^1^ ^,2^).

### Spectral Library Generation

The DDA data were processed using the Proteome Discoverer (Thermo Scientific, Germany) software, and searched against the human SwissProt database appended with the iRT fusion protein sequence (Biognosys). The search allowed two missed cleavage sites in the trypsin digestion, cysteine carbamidomethylation was set as a fixed modification, parent ion mass tolerances were set to 10 ppm, and fragment ion mass tolerances were set to 0.02 Da. The applied false discovery rate (FDR) cutoff was 0.01 at the protein level. The results were imported to the Spectronaut Pulsar (Biognosys, Switzerland) software to generate the library.

### PRM Mass Spectrometry

The PRM analysis was performed on TripleTOF 5600 +. The separation of the peptides was performed on an RPC18 self-packing capillary LC column (75 μm × 100 mm, 3 μm). The eluted gradient was 5–30% buffer B1 (0.1% formic acid, 99.9% ACN; flow rate, 0.3 μl/min) for 60 min. For ionization, a spray voltage of 2.10 kV and a capillary temperature of 60°C were used. The peptides (Supplementary Table S3) were monitored using the PRM acquisition mode for performing MS/MS scans of the precursor ions for the all peptide markers along the complete chromatographic run, and each sample was run for two times. The normalized collision energy was fixed to 35%, and the accumulated time was 300 s.

### Data Analysis

The DIA-MS data were analyzed using the Spectronaut Pulsar (Biognosys, Switzerland) with default settings. In brief, the retention time prediction type was set to dynamic iRT. Interference correction on the MS2 level was enabled. Peptide intensity was calculated by summing the peak areas of their respective fragment ions for MS2, and the protein intensity was calculated by summing the intensity of their respective peptides. Cross run normalization was enabled to correct for systematic variance in the LC-MS performance, and local normalization strategy was used. The normalization was based on the assumption that, on average, a similar number of peptides was upregulated and downregulated, and the majority of the peptides within the sample were not regulated across runs, and along the retention time. Protein inference, which gave rise to the protein groups, was performed on the principle of parsimony using the ID picker algorithm, as implemented in Spectronaut. All results were filtered by a Q value cutoff of 0.01 (corresponds to an FDR of 1%).

For the PRM mode, Skyline software (version 3.5.0.9319) was used for the selection of the suiTable m/z precursor ion → m/z fragment ion transition for the selected candidate peptide biomarkers. Peptide settings: the enzyme was set as trypsin [KR/P], and the maximum number of missed cleavages was set as 2. The peptide length was set as 8–25; the variable modifications were set as carbamidomethyl on Cys and oxidation on Met; and the maximum number of variable modifications was set as 3. Transition settings: the precursor charges were set as 2 and 3; the ion charges were set as 1 and 2; the ion types were set as b, y, and p. The product ions were set to range from ion 3 to the last ion, and the ion match tolerance was set as 0.02 Da.

### Rank the Abundance

In order to rank the relative abundance of different proteins, an intensity-based absolute quantification (iBAQ) algorithm was used (Schwanhausser et al., 2011). Protein intensities summarizing all of the identified peptide intensities were constructed using Progenesis LC-MS (version 2.6, Non-linear Dynamics, United Kingdom), according to a previously described procedure (Hauck et al., 2010). The iBAQ value was obtained by peptide intensities divided by the number of theoretically observable peptides of the protein (calculated by *in silico* protein digestion; all fully tryptic peptides between 6 and 30 amino acids were counted) (Liu et al., 2017). The relative iBAQ intensities were computed by dividing the absolute iBAQ intensities by the sum of all absolute iBAQ intensities. The estimated protein abundances were calculated by multiplying the relative iBAQ intensities by the molecular weight of the protein.

### Bioinformatics Analysis

All differentially expressed proteins were assigned to their gene symbol according to the Panther database^3^. Protein classification was performed based on functional annotations using Gene Ontology (GO) for biological processes, molecular function, and cellular component categories.

The investigators uploaded the information of the differently expressed proteins (DEPs) and validated the DEPs to the STRING database^4^ for protein–protein interaction (PPI) network analysis, and the minimum interaction score was set at 0.4. The biomolecular interaction networks are instructed within the software Cytoscape (version 3.7.1) and its plugins (Chin et al., 2014).

For the Ingenuity Pathway Analysis (IPA), the SwissProt accession numbers were uploaded to the IPA software (Ingenuity Systems, Mountain View, CA, United States). The proteins were mapped to the disease and function categories, and canonical pathways available in ingenuity, and other databases that were ranked by the *P*-value.

## Results

### Workflow

In the present study, 21 cataract AH samples were used to generate a comprehensive profile of the human AH proteome. All AH samples were pooled into one sample. The pooled sample was digested and analyzed by 2D-RP-RP LC-MS/MS. The functional annotation was provided by the GO annotation and IPA analysis.

**FIGURE 1:**
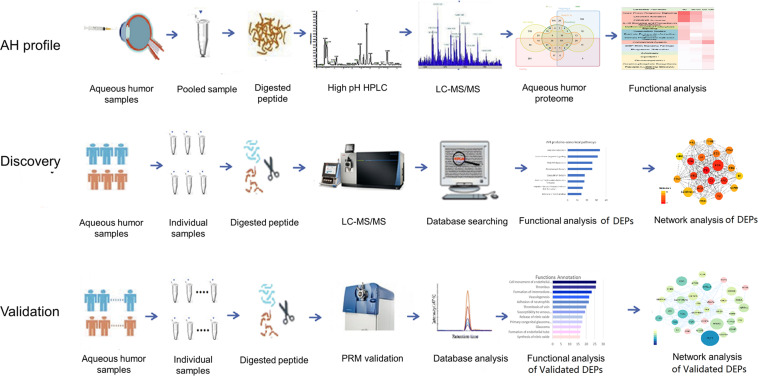
Workflow of the human aqueous humor (AH) fluid proteome profile analysis and the discovery/validation of differently expressed proteins (DEPs) in the neovascular glaucoma (NVG) treatment.

In the disease AH proteomic analysis, six AH samples from three NVG patients before and after drug treatment were analyzed using the DIA technique. After functional analysis, the key differential proteins were validated by PRM technology in an independent group (14 samples) (Figure 1).

### Database: A Comprehensive Profile of the AH Proteome

**FIGURE 2:**
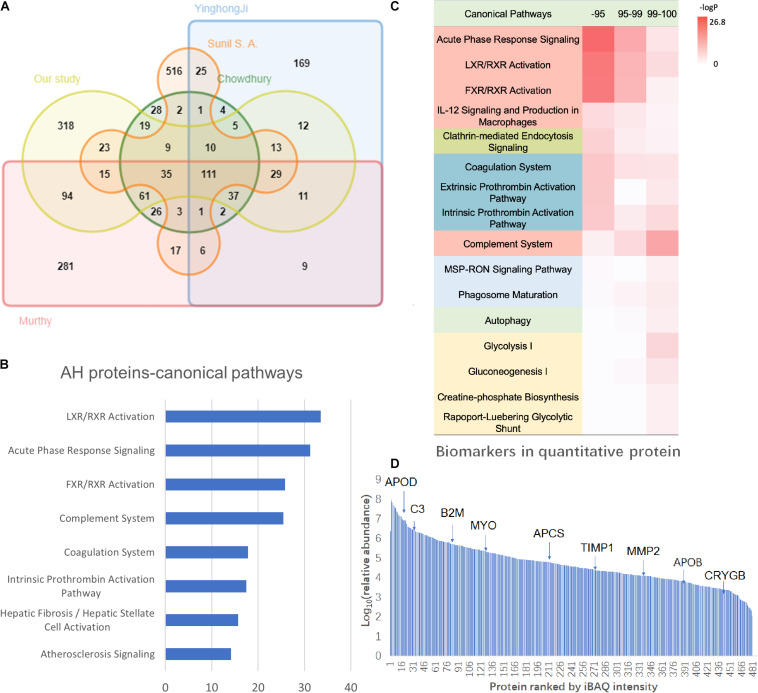
The AH proteome analysis of the present study. **(A)** The Venn diagram of the identified proteins and comparison of the present results with the previous AH proteome data. **(B)** The Ingenuity Pathway Analysis (IPA) canonical pathway analysis of the AH proteome. **(C)** The hierarchical clustering based on the intensity-based absolute quantification (iBAQ) quantification and IPA annotation. **(D)** The dynamic range of the AH proteome with key biomarkers marked. The *X*-axis indicates the protein numbers ranked by the iBAQ intensity, and the *Y*-axis indicates the log10 (relative intensity) of the proteins.

In the pooled sample, through the 2D-LC/MS/MS analysis of 5,368 peptides, 802 proteins were identified (431 proteins with at least two unique peptides) (detailed data in Supplementary Table S2). The investigators compared the present results with those in previous AH proteome studies (Table 1). Most proteins in the present study (60.3%) overlapped with those in previous studies, and 318 proteins (39.7%) were newly identified. In the present study offline, high-pH RPLC separation and online low-pH RPLC MS were used, which could achieve higher proteomic separation efficiency. In addition, a new state-of-the-art mass spectrometer, Orbitrap Fusion Lumos, was used to acquire the MS/MS data, which could provide faster scan speed and higher detection sensitivity. Therefore, 318 proteins could be newly found in the AH proteome. Taking all the results of the AH proteome into one database, the human AH proteomes were expanded to 1,888 (Figure 2A, detailed data in Supplementary Table S3).

**TABLE 1:** Comparison of 5 aqueous humor (AH) proteome studies.

**Year**	**Author**	**Disease**	**Method**	**Instrument**	**Filter**	**Number of proteins identified**	**References**
2010	Chowdhury et al.	Cataracts	Nano-LC-ESI-MS/MS	ThermoFinnigan LTQ Orbitrap Hybrid	At least 2 unique peptides	354	Chowdhury et al., 2010
2015	Krishna R. Murthy et al.	Cataracts	LC-MS/MS	LTQ-Orbitrap Velos mass spectrometer	FDR < 1%	737	Murthy et al., 2015
2015	YinghongJi et al.	Cataracts	iTRAQ LC–MS/MS	Orbitrap QExactive mass spectrometer	FDR < 1%	445	Ji et al., 2015
2019	Sunil S. Adav et al.	Cataracts	LC-MS/MS	Q-Exactive instrument	FDR < 1%	816	Adav et al., 2019
2019	This study	Cataract	LC-MS/MS	Orbitrap Fusion Lumos tribrid	FDR < 1%	802	–

The GO analysis of the AH proteome was performed using the PANTHER classification system (see text footnote 1). In the cellular component category, the AH matrices were enriched in the extracellular region. The main molecular functions of the AH protein were binding and catalytic activity. AH proteins principally participate in the metabolic and biological regulation process (Supplementary Figure S1).

The IPA pathway analysis revealed that these proteins are primarily involved in immune/inflammatory pathways (LXR/RXR activation, acute phase response signaling, FXR/RXR activation, and complement system), coagulation-related pathways (coagulation system, intrinsic prothrombin activation pathway, extrinsic prothrombin activation pathway, GP6 signaling pathway, and atherosclerosis signaling), and energy metabolism-related pathways (glycolysis I, gluconeogenesis I, and glucocorticoid receptor signaling) (Figure 2B, detailed data in Supplementary Table S4). Proteins related to immunity and inflammation may reflect the particularity of ocular immune privilege (Streilein, 2003).

In order to further understand the AH proteome, the quantitative proteomic analysis was provided by the iBAQ algorithm (Schwanhausser et al., 2011). In this study, 480 proteins were quantified, and the dynamic range of relative abundance spanned 7 orders of magnitude (detailed data in Supplementary Table S2). In addition, the investigators ranked these 480 quantitative proteins according to their abundance, in which high abundance proteins were the top 95% proportion (47 proteins), medium abundance ones were within the 95–99% proportion (87 proteins), and low abundance ones were 1% of the proportion (346 proteins) (detailed data in Supplementary Table S4). The canonical pathway analysis revealed the medium and high-abundance proteins involved in similar pathways, such as the inflammatory/immune-related pathways (acute phase response signaling, LXR/RXR activation, FXR/RXR activation, IL-12 signaling, and production in macrophages) and coagulation-related pathways (coagulation system, extrinsic prothrombin activation pathway, intrinsic prothrombin activation pathway). Obviously different from high-abundance/medium-abundance proteins, low-abundance proteins are involved in the complement pathway, energy metabolism pathways (glycolysis I, gluconeogenesis I, creatine-phosphate biosynthesis, Rapoport–Luebering glycolytic shunt), and phagocytosis-related pathways (MSP-RON signaling pathway, phagosome maturation) (Figure 2C).

As shown in Table 2 the 10 most abundant protein assemblages accounted for approximately 78% of the total AH proteins. All of these proteins were found in the blood^5^ (Bastian et al., 2008). One of the most abundant protein in the AH is the receptor-type tyrosine-protein phosphatase zeta (PTPRZ1). PTPRZ1 is also known as RPTPβ/ζ, RPTPβ, or RPTPζ. The expression of PTPRZ1 is restricted to the central nervous system and localizes to glial cells. This may be involved in manifold recognition events in the construction of neural networks (Bouyain and Watkins, 2010). Evidence indicates that RPTPβ/ζ may play an important role in establishing the phenotype differentiation of Müller glia cells. It is a critical signaling molecule in the developing mature mouse retina (Horvat-Brocker et al., 2008). Other high-abundance proteins, such as transthyretin (TTR) and transferrin (TF), were also reported to play roles in pathological or physiological processes of the eye (Picard et al., 2015; Daruich et al., 2019).

**TABLE 2:** The 10 most abundant proteins in AH.

**Accession**	**Description**	**Gene names**	**iBAQ**	**Percentage**
P23471	Receptor-type tyrosine-protein phosphatase zeta	PTPRZ1	6.79E + 07	29.60%
P02768	Serum albumin	ALB	2.30E + 08	27.34%
P02787	Serotransferrin	TF	4.99E + 07	6.59%
P01859	Immunoglobulin heavy constant gamma 2	IGHG2	7.07E + 07	4.35%
P08185	Corticosteroid-binding globulin	SERPINA6	4.29E + 07	3.31%
P01834	Immunoglobulin kappa constant	IGKC	9.58E + 07	1.93%
P07288	Prostate-specific antigen	KLK3	3.55E + 07	1.75%
P41222	Prostaglandin-H2 D-isomerase	PTGDS	3.65E + 07	1.32%
P02763	Alpha-1-acid glycoprotein 1	ORM1	2.64E + 07	1.06%
P02766	Transthyretin	TTR	2.18E + 07	0.59%

According to the IPA annotation, 218 proteins are found to be potential biomarkers for ophthalmology diseases (Figure 2D). Particularly, many were reported to be crucial factors in glaucoma pathological features (detailed data in Supplementary Table S2). C3 reduction was associated with the severity of glaucomatous optic nerve degeneration (Bosco et al., 2018). The increase in TIMP1 and TIMP2 may lead to the inhibition of MMP2 activity and contribute to the IOP of primary open-angle glaucoma (Ashworth Briggs et al., 2015). In addition, MYOC is generally considered to be closely correlated to the occurrence and development of glaucoma (Nazir et al., 2018). In addition to glaucoma, these AH proteins have also been reported to be associated with a variety of other common ophthalmology diseases, such as age-related macular degeneration (Cashman et al., 2011) and cataract (Shiels and Hejtmancik, 2015).

### The Differential AH Proteomic Analysis of NVG in Conbercept Treatment

**FIGURE 3:**
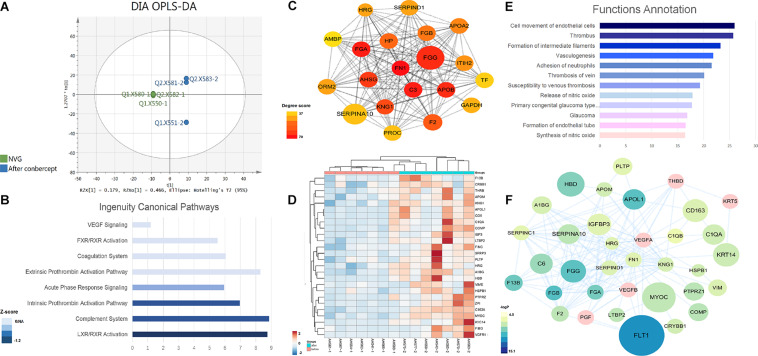
The DEP analysis of the AH proteome in the NVG treatment with conbercept. **(A)** The orthogonal partial least squares discriminant analysis (OPLS-DA) score plot based on the DIA data of the AH proteome from NVG patients (green), and after conbercept treatment patients (blue). The mean center-scaling model (*P* = 1.54857e-07). **(B)** The IPA ingenuity canonical pathway analysis of DEPs. **(C)** The subnetwork of DEPs with high node degree. **(D)** The hierarchical clustering based on the PRM data of the validated DEPs. **(E)** The function annotation of validated DEPs. **(F)** The network of validated proteins.

A total of 541 proteins were identified by the DIA method in AH samples before and after conbercept treatment (Supplementary Table S5). Apparent differences between before and after the conbercept treatment of samples were observed from the OPLS-DA score plot (*P* = 1.54857e-07) (Figure 3A). Among the 541 proteins, 254 proteins with a fold change of >1.5 were defined as differentially expressed proteins (DEPs) (190 upregulated DEPs, 64 downregulated DEPs).

The GO annotation was analyzed according to their molecular function, biological progress, and cellular component (Supplementary Figure S2). DEPs are basically located in the extracellular region, while these mainly participate in the cellular process, and the function of DEPs is enriched in the binding and catalytic activity. The IPA analysis suggested that eight pathways were significantly involved (Figure 3B). Among these, the LXR/RXR activation, complement system, intrinsic prothrombin-activation pathway, and acute phase response signaling are significantly suppressed.

The PPI network was constructed in cytoscape. The network with 178 DEPs centered on FN1 (Figure 3C). The cytoHubba plugin was used to find the hub proteins (Chin et al., 2014). The top node degree fibronectin 1 (FN1) was reported to be involved in the pathogenesis of glaucoma (Feng and Xu, 2019). It was found that FN1 interacted with the DEPs and mainly participated in the angiogenesis and coagulation cascades pathway. In addition, the expanded subnetwork of these proteins centered on VEGF, which is the key target of the NVG treatment. These above key proteins were used for the PRM validation.

Through PRM analysis, 33 peptides that corresponded to 26 proteins (all upregulated) (Figure 3D and Table 3) were validated. The IPA analysis of the validated DEPs suggested that thrombosis, angiogenesis, and the formation/release of nitric oxide, as well as the variety types of glaucoma pathways, were significantly involved (Figure 3E, detailed data in Supplementary Table S6). Compared to the main pathways of the whole DEPs, these pathways are more directly correlated to the pathological changes of NVG. The PPI network of validated DEPs was also constructed (Figure 3F). These validated DEPs with rich interactions, formed a network centered on VEGFA, VEGFB, and FN1, with a reasonable result, given that the VEGF receptor is the main composition of conbercept (Wu et al., 2013).

**TABLE 3:** The differently expressed proteins (DEPs) (26) that were validated using PRM technology (all up regulated).

**Protein name**	**Accession**	**Gene symbol**	**FC (test) after treatment/neovascular glaucoma (NVG)**	**FC (validation)after treatment/NVG**	***P*-value**
Insulin-like growth factor-binding protein 3	P17936	IGFBP3	1.66E + 00	3.99E + 00	0.019545
Complement C1q subcomponent subunit A	P02745	C1QA	2.50E + 00	3.01E + 00	0.045073
Apolipoprotein L1	O14791	APOL1	1.50E + 00	2.40E + 00	0.027965
Prothrombin	P00734	F2	1.77E + 00	2.48E + 00	0.043172
Latent-transforming growth factor beta-binding protein 2	Q14767	LTBP2_HUMAN	2.35E + 00	7.81E + 00	0.023327
Apolipoprotein M	O95445	APOM	5.31E + 00	4.69E + 00	0.013478
Alpha-1B-glycoprotein	P04217	A1BG	1.81E + 00	1.84E + 00	0.002988
Vimentin	P08670	VIM	1.56E + 00	1.84E + 00	0.037981
Fibrinogen gamma chain	P02679	FGG	9.24E + 00	3.95E + 00	0.013217
Kininogen-1	P01042	KNG1	2.46E + 00	2.91E + 00	0.010031
Secreted frizzled-related protein 3	Q92765	FRZB	1.89E + 00	1.83E + 00	0.013319
Fibronectin	P02751	FN1	1.93E + 00	1.56E + 00	0.012816
Hemoglobin subunit delta	P02042	HBD	1.89E + 00	1.96E + 00	0.00736
Keratin, type I cytoskeletal 14	P02533	KRT14	7.22E + 00	2.15E + 00	0.035922
Phospholipid transfer protein	P55058	PLTP	1.52E + 00	3.54E + 00	0.001783
Histidine-rich glycoprotein	P04196	HRG	2.65E + 00	2.54E + 00	0.020115
Heat shock protein beta-1	P04792	HSPB1	1.56E + 00	1.54E + 00	0.014324
Coagulation factor XIII B chain	P05160	F13B	2.46E + 00	1.77E + 00	0.018517
Complement component C6	P13671	C6	9.48E + 00	3.19E + 00	0.028325
Scavenger receptor cysteine-rich type 1 protein M130	Q86VB7	CD163	3.91E + 00	3.18E + 00	2.95E-05
Vascular endothelial growth factor receptor 1	P17948	FLT1	2.03E + 00	4.26E + 00	0.043476
Receptor-type tyrosine-protein phosphatase zeta	P23471	PTPRZ1	2.56E + 00	2.60E + 00	0.036656
Cartilage oligomeric matrix protein	P49747	COMP	1.66E + 00	2.24E + 00	0.019845
Beta-crystallin B1	P53674	CRYBB1	2.51E + 00	2.04E + 00	0.014417
Myocilin	Q99972	MYOC_HUMAN	3.33E + 03	2.11E + 01	0.005214
Protein Z-dependent protease inhibitor	Q9UK55	SERPINA10	1.95E + 00	3.16E + 00	0.001224

## Discussion

In the present study, a total of 802 proteins were identified in the AH proteome, and the current data provided a baseline proteomic profile of human AH. Furthermore, through the differential analysis of AH proteome in the NVG treatment, 26 DEPs were validated. These proteins may be the crucial factors in the NVG pathological process.

### AH Proteome Function Analysis

#### Comparison of AH and Plasma Proteome

The source of AH proteins has been discussed for years; the plasma-derived protein could diffuse from the ciliary body stroma (Freddo, 2013), and the additional source of proteins in AH is the ciliary body itself (Coca-Prados and Escribano, 2007).

To date, in human AH proteome data (1,888 in total), 825 (43.7%) proteins are in common with the plasma proteome. In order to understand the proportion of AH proteins, the investigators calculated these in quantitative proteins. Common proteins accounted for 91.2% of the total AH proteome abundance, indicating that the main components of the AH proteome come from plasma. Albumin is the most abundant protein in AH and plasma proteome. Albumin plays an important role in the delivery of long-chain fatty acids, vitamins, and hormones to cells in many tissues of the body. It is demonstrated that this physiologically critical macromolecule from the aqueous humor can facilitate the delivery of important metabolites into the lens (Sabah et al., 2004). Additionally, the abundance of receptor-type tyrosine-protein phosphatase zeta, serotransferrin, corticosteroid-binding globulin, semenogelin-1, fibrinogen alpha chain, and vitamin D-binding protein in AH is relatively high.

**FIGURE 4:**
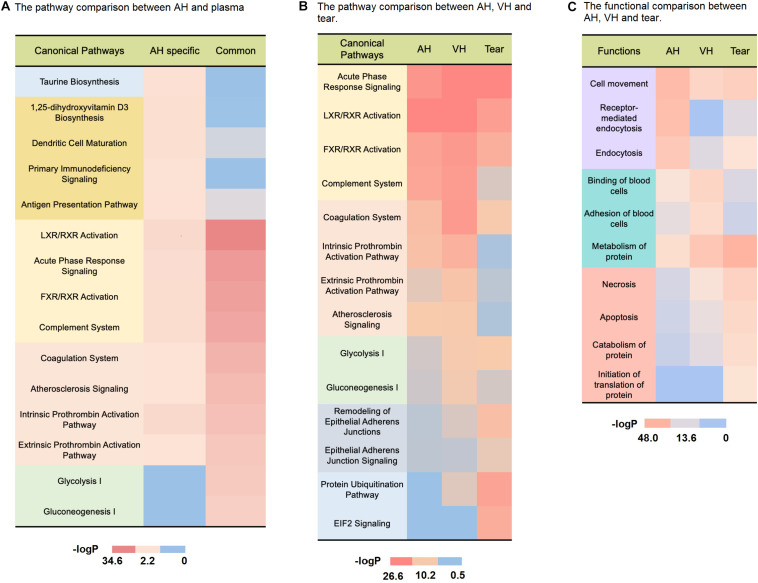
Comparison of the AH and body fluid proteome. **(A)** The pathway comparison between AH-specific proteins and plasma-derived proteins. **(B)** The pathway comparison between AH, VH, and tears. **(C)** The functional comparison between AH, VH, and tears.

Common proteins are mostly enriched in immune/inflammatory-related pathways (complement system, LXR/RXR activation, acute phase response signaling, FXR/RXR activation) according to pathway analysis (Figure 4A, detailed data in Supplementary Table S7), and the proteins involved account for 30% of all quantitative protein abundance. It has been recognized that the major component of immune privilege, termed anterior chamber-associated immune deviation (ACAID), is complement dependent (Sohn et al., 2003). A fine balance between complement activation and suppression is important for maintaining a healthy environment within the eye. Complement disorder status could contribute to several eye diseases, including glaucoma, diabetic retinopathy, and age-related macular degeneration (AMD) (Miyahara et al., 2003; Ghosh et al., 2015; Clark and Bishop, 2018). In addition, common proteins were also involved in coagulation-related pathways (coagulation system, atherosclerosis signaling, intrinsic prothrombin activation, extrinsic prothrombin pathway), which suggested that coagulation could be an important function of AH and plasma.

It was also found that there were 1,063 (56.3%) AH-specific proteins, which accounted for 8.8% of the total AH proteome abundance. Most of these were low-abundance proteins. The pathway analysis revealed that AH-specific proteins were enriched in taurine biosynthesis, 1,25-dihydroxyvitamin D3 biosynthesis (Figure 4A, detailed data in Supplementary Table S7). Taurine, which is well known as a common ingredient of energy drinks, is the most abundant amino acid in the retina, vitreous, lens, cornea, iris, and ciliary body. It is essential to the development of the nerve system and the maintenance of normal eye physiological activity (Oja and Saransaari, 2017; Pardue and Allen, 2018). The 1,25-dihydroxyvitamin D3 can modulate inflammatory responses in the eye (Lemire, 1995). In fact, it has been demonstrated that the topical administration of 1,25-dihydroxyvitamin D3 inhibits Langerhans cell migration from the central cornea, corneal neovascularization, and the production of cytokines (i.e., interleukin-1-6-8) in experimental animals (Nebbioso et al., 2017). Moreover, many AH-specific proteins were correlated with immune activities (dendritic cell mutation, primary immunodeficiency signaling, antigen presentation pathway) as shown in Figure 4A. Dendritic cells are the most efficient antigen-presenting cells (Théry and Amigorena, 2001). Tolerance-promoting antigen-presenting cells have been recognized as an essential part of eye immune privilege (Streilein, 2003).

#### Comparison of AH, Vitreous Humor, and Tear Proteome

Vitreous humor is a transparent medium that functions to maintain the shape of the eye and transport nutrients. As an important component of the innate defense system of the eye, tear provides protection against a range of potential pathogens. In order to analyze the function of different ocular fluids, the investigators compared the whole human AH data with vitreous humor (Murthy et al., 2014) and tear (Dor et al., 2019) from other ocular proteomic studies.

As shown in Figures 4B,C, compared to tear, AH was more functionally related to VH. In ocular circulation, it is the continuous flow of AH that nourishes the avascular cornea, lens, and vitreous compartment (Rocha et al., 2014). Though it is widely held that the fluid in the VH is stagnant, recent research of David W. Smith indicated that aqueous fluid could transport through the vitreous (Smith et al., 2020). Our proteome analysis also suggested there could be more frequent molecular exchanges between them.

In the pathway analysis shown in Figure 4B, the protein pathways of three ocular fluids were aggressively concentrated on the immune/inflammation-related activity (detailed data in Supplementary Table S7). As a photosensitive organ, the eye has unique immunologic properties. Inflammation and oxidative damage can have a devastating impact on the light transmittance of the anterior chamber and vitreous body. The transforming growth factor-β (TGFB), α-melanocyte-stimulating hormone (MSH) in AH and vitreous humor can suppress the expression of delayed-type hypersensitivity responses (Niederkorn, 2003). These multiple immune-related proteins help light transmittance eye fluids maintain an operational condition for the immune privilege and make the eye free from immune-related damage. Tear is enriched in antimicrobial proteins, such as lactoferrin (LTF) and lysozyme (LYZ). These can maintain an effective immune defense at the ocular surface to prevent infections (Hanstock et al., 2019).

In the functional analysis in Figure 4C, compared to other ocular fluids, AH proteins were remarkably involved in the endocytosis and clathrin-mediated endocytosis process (detailed data in Supplementary Table S7). The dendritic cells (DCs) and macrophages in the eye are more immature. Therefore, their phagocytic function tends to be antigen capture, rather than antigen presentation (McMenamin, 1999), thereby limiting the exposure of the anterior chamber of the eye to exogenous antigens and avoiding excessive immune response. Accumulated excess pigment debris could potentially disrupt the clear visual axis or break the outflow homeostasis of AH (Gagen et al., 2013), thereby elevating the IOP (Dang et al., 2018). The phagocytosis of melanin granules is critical to keep excess pigment granules from distributing to the AH (Chinnery et al., 2017). In accordance with this, it can be easily recognized in Figure 4A that the AH proteins are active in antigen presentation and dendritic cell-related pathways.

The pathway analysis revealed that vitreous humor proteins participated more in energy metabolism than other ocular fluids (Figure 4B, detailed data in Supplementary Table S7). The vitreous humor fills the posterior segment of the eye between the lens and retina in vertebrates (Sebag, 1992). The retina has the largest oxygen consumption by weight of any tissue in the human body (Wong-Riley, 2010). As an adjacent tissue of the retina, nourishing the retina is the main function of vitreous humor, and vitreous humor proteins enriched in energy metabolism can meet the high-energy requirements of retinal neurons. Additionally, as shown in Figure 4B, VH was more involved in the coagulation progress (intrinsic prothrombin activation, extrinsic prothrombin activation atherosclerosis signaling pathways) than AH and tear. It is reported that thrombin dysregulation may cause proinflammatory and profibrotic mediator production by retinal pigment epithelial cells and thrombin, and Factor Xa may play a role in vitreoretinal disorders such as proliferative vitreoretinopathy, proliferative diabetic retinopathy, and exudative age-related macular degeneration (Bastiaans et al., 2013). Furthermore, VH was also involved in binding and adhesion of blood cells. Its mechanism required further investigation.

Different from AH and VH, tear proteins were basically enriched in protein metabolism, necrosis, apoptosis functions, and the remodeling of epithelial adherents’ junction EIF2 signaling and protein ubiquitination pathways (Figures 4B,C, detailed data in Supplementary Table S7). The necrosis- and apoptosis-related proteins in tears may indicate the frequent host–pathogen interaction in tears (Sridharan and Upton, 2014). Ubiquitination has a crucial role in the regulation of immune tolerance (Hu and Sun, 2016). Ubiquitination-related proteins in the eye show selectivity toward oxidatively modified proteins (Shang and Taylor, 2004). Through removing oxidatively damaged proteins and protein fragments, the tear might alleviate the accumulation of cytotoxicity in the ocular surface. Tear proteins reside in the ocular surface. As the front line of eye defense, they provide protection from noxious chemicals and pathogens to the avascular cornea (Green-Church et al., 2011). It has been reported that some tear components can prevent epithelial cell invasion and promote the epithelial expression of innate defense molecules (McDermott, 2013). Besides, EIF-2A is one of the key regulators of the integrated stress response, which is a common adaptive pathway that is activated in response to diverse stress stimuli, thus, restoring cellular homeostasis (Martina et al., 2016). It is reported that air exposure-induced autophagy is accompanied by the increase in phosphorylated EIF-2A, which is indispensable for the maintenance of corneal epithelial physiology and cell survival (Wang et al., 2019).

### Changes in AH Proteome in NVG Treatment

In order to determine whether the human AH proteome could reflect the pathological alternation in NVG treatment, the investigators used the DIA and PRM techniques to find and verify the DEPs. The function of 26 validated DEPs was focused on angiogenesis, vasculogenesis, etc.

The PPI network suggested that 26 upregulated DEPs have significant interactions, and most members of this network, such as FLT1, IGFBP3, PTPRZ1, FN1, and KNG1, are involved in the angiogenesis function in IPA analysis. As the first neighbors of VEGFA, these have direct connections with vascular endothelial growth in NVG.

FLT1 (vascular endothelial growth factor receptor 1), as a cell-surface receptor for VEGFA, VEGFB, and PGF, plays an essential role in the development of embryonic vasculature and the regulation of angiogenesis (Melincovici et al., 2018). FLT1 has 10 times higher affinity for VEGF than VEGFR-2, and has a lower tyrosine kinase activity (Hoeben et al., 2004). It can make VEGF (the key target in NVG treatment) less accessible for VEGFR-2 and has a “negative role” in vasculogenesis. A soluble form of FLT1 can reduce the amount of VEGFs available for the interaction with their transmembrane receptors, thereby negatively regulating the VEGFR-mediated signaling (Failla et al., 2018).

In addition to vascular-related functions, the number of thrombus coagulation processes was involved. In the validated DEPs, F13B (also known as coagulation factor XIII B chain) participates in susceptibility to venous thrombosis. In the present study, the increase in F13B may indicate the release of the hypercoagulated state of the microvasculature of the eye. Deng et al. (2019) observed that the mean flow area of the choriocapillaris significantly improved after conbercept treatment in patients. F13B comprises of the inactive form of transglutaminase, a blood coagulation factor. This participates in the process of cross-linking between fibrin molecules and contributes to the stabilization of clot formation (Bagoly et al., 2012). The visual recovery of patients after conbercept treatment was observed in macular edema secondary to branch retinal vein occlusion patience (Wang et al., 2020). Although there is no direct experimental evidence that supports that F13B plays a role in the regeneration of the human optic nerve, previous data have shown that the transient increase in F13B in retinal ganglion cells (RGCs) promotes neurite sprouting from injured RGCs (Sugitani et al., 2012), while the sustained increase in F13B in optic nerves facilitates neurite elongation from axon regeneration.

## Conclusion

The AH proteome is an important potential source of biomarkers for identifying posterior pathophysiological changes. This work can provide a baseline reference for further AH proteomic analysis and contribute to the application of AH proteome in ophthalmology disease. With the advances in mass spectrometry instrumentation, proteomic methodologies, and bioinformatics, the proteomic tool would dramatically transform the approach to the treatment of eye diseases in the future.

## Data Availability Statement

The datasets presented in this study can be found in online repositories. The names of the repository/repositories and accession number(s) can be found in the article/Supplementary Material.

## Ethics Statement

The studies involving human participants were reviewed and approved by The Institutional Review Board of The Institute of Basic Medical Sciences (Beijing, China). The patients/participants provided their written informed consent to participate in this study.

## Author Contributions

WS, YW, and CH conceived and designed the study. WS and CH supervised the study. MY drafted the manuscript. MY, FX, and WS wrote, reviewed, and edited the manuscript. CH acquired the funding. WS and FX coordinated the project. MY, XianL, and WL performed the experiments. MY and FX analyzed the data. XianL, FX, and HS performed the validation experiments. MY, XiaoL, and HS interpreted the data. MY and XiaoL drew the figures. YW and ZG provided the resources. All authors contributed equally and have read and approved the final manuscript.

## Conflict of Interest

XianL was employed by company Shanghai AB Sciex Analytical Instrument Trading Co., Ltd. The remaining authors declare that the research was conducted in the absence of any commercial or financial relationships that could be construed as a potential conflict of interest.

## Footnotes

1 http://www.iprox.org

2 https://www.iprox.org/page/PSV023.html;?url=1595559292439gM2p, password: 6uw9.

3 http://www.pantherdb.org/

4 https://string-db.org/

5 https://bgee.org/

## Supplementary Material

TABLE S1 Sheet 1. Detailed clinical information of AH samples. Sheet 2. Detailed clinical information of NVG samples before and after treatment.

TABLE S2 Detailed IBAQ value, unique peptide, biomarker etc. information of all identified AH proteins and their abundance group.

TABLE S3 Detailed AH protein list of 4 previous studies and our study.

TABLE S4 Sheet 1. Ingenuity pathway analysis results of AH proteins. Pathways ranked by -log(10)P-value. Sheet 2. Functions of AH proteins ranked by -log(10)P-value. Sheet 3. Main functions of different abundance group of AH proteins.

TABLE S5 Detailed data of DIA analysis of NVG samples before and after treatment.

TABLE S6 Detailed PRM information of selected DEPs.

TABLE S7 Sheet 1. Proteins list of whole human AH, vitreous humor, tear and plasma. Sheet 2. Pathway scores (the –log10 of the Fisher’s Exact Test *p*-value) of AH specific proteins and AH proteins in common with plasma. Sheet 3. Pathway score (the –log10 of the Fisher’s Exact Test *p*-value) comparison of three ocular fluids. Sheet 4. function score (the –log10 of the Fisher’s Exact Test *p*-value) comparison of three ocular fluids.
