# Supplementary figures and images for: Proteomic Study of Aqueous Humor and Its Application in the Treatment of Neovascular Glaucoma
Source: Front Mol Biosci. 2020 Oct 8;7:587677. doi: 10.3389/fmolb.2020.587677 (PMC7580691; doi:10.3389/fmolb.2020.587677)

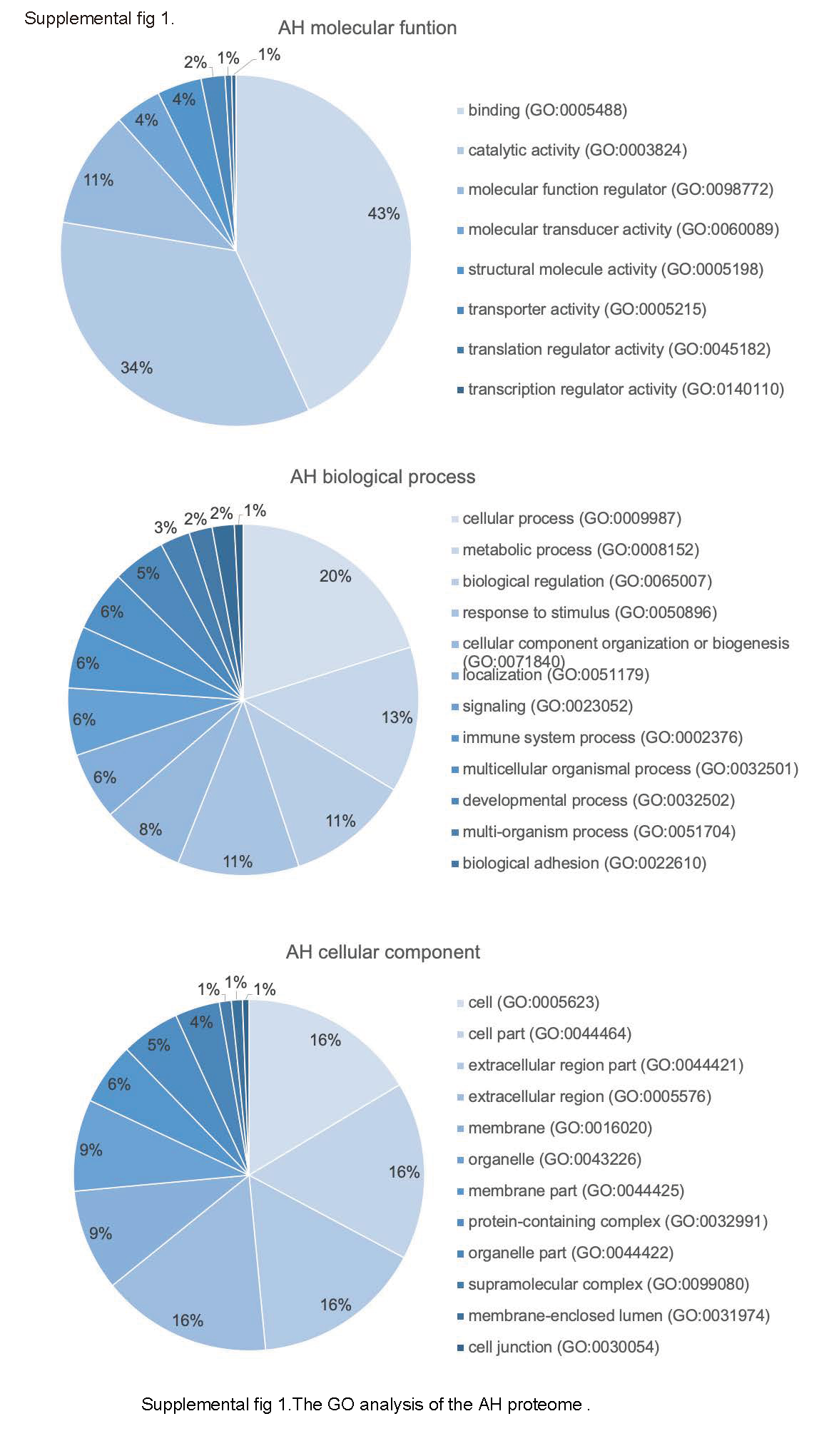

Supplement: Supplementary file 7 [file Image_1.TIF]

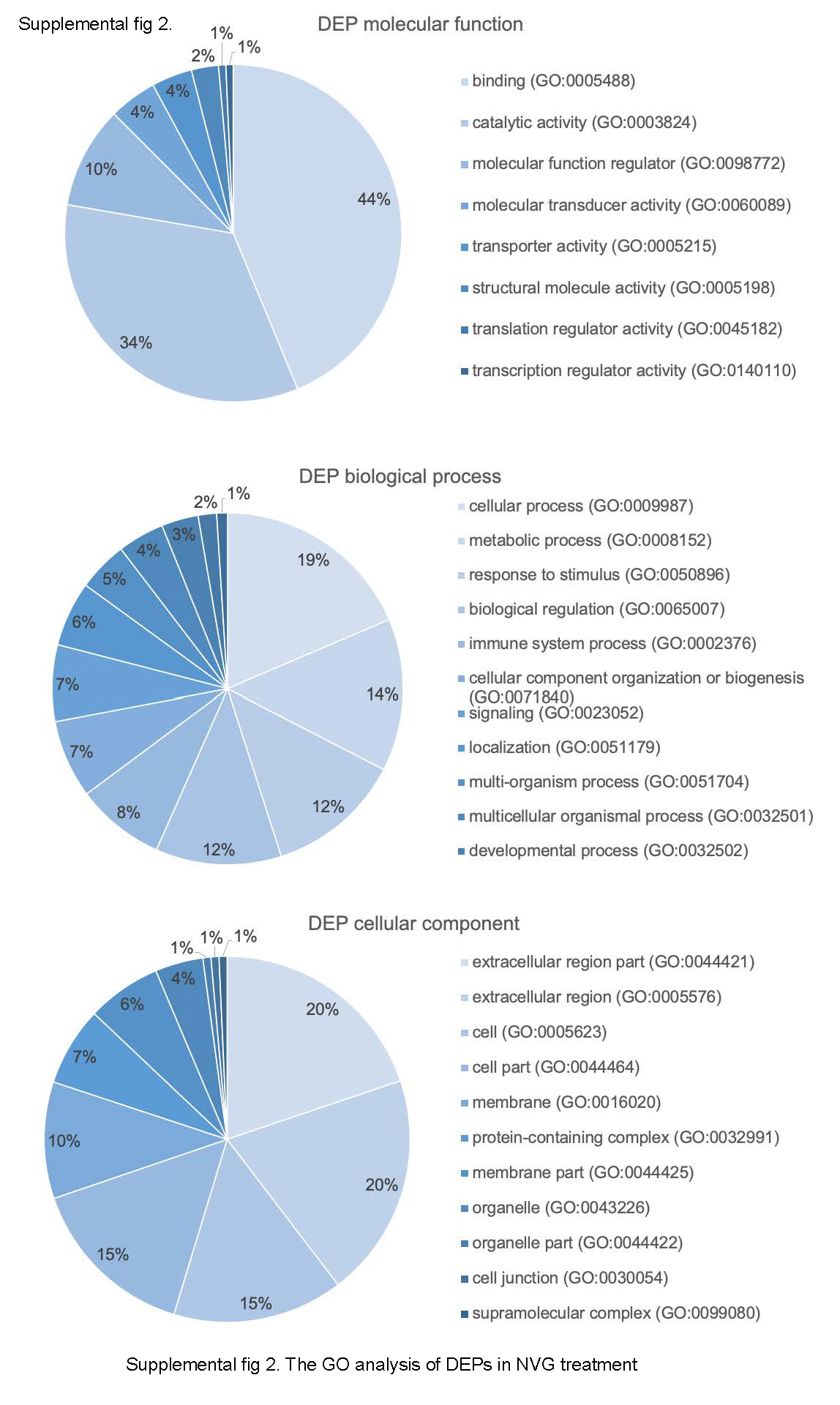

Supplement: Supplementary file 8 [file Image_2.TIF]
